# Evaluation of the design and structure of electronic medication labels to improve patient health knowledge and safety: a systematic review

**DOI:** 10.1186/s13643-023-02413-z

**Published:** 2024-01-02

**Authors:** Sara Saif, Tien Thi Thuy Bui, Gyana Srivastava, Yuri Quintana

**Affiliations:** 1 Belmont University College of Pharmacy, Nashville, TN 37212 USA; 2 Division of Clinical Informatics, Beth Israel Deaconess Medical Center, Boston, MA 02215 USA; 3 Massachusetts College of Pharmacy, 179 Longwood Ave, Boston, MA 02115 USA

## Abstract

**Introduction:**

Patient misunderstanding of instructions on medication labels is a common cause of medication errors and can result in ineffective treatment. One way to better improve patient comprehension of medication labels is by optimizing the content and display of the information.

**Objectives:**

To review comparative studies that have evaluated the design of a medication label to improve patient knowledge or safety.

**Methods:**

Studies were selected from systematic computerized literature searches performed in PubMed, Embase (Elsevier), Cochrane Central (EBSCO), Cumulative Index to Nursing and Allied Health Literature-CINAHL (EBSCO), and Web of Science (Thomson Reuters). Eligible studies included comparative studies that evaluated the design of a medication label to improve patient knowledge or safety.

**Results:**

Of the 246 articles identified in the primary literature search, 14 studies were selected for data abstraction. Thirteen of these studies significantly impacted the patient understanding of medication labels. Three studies included a measure of patient safety in terms of medication adherence and dosing errors. The utilization of patient-centered language, pictograms/graphics, color/white space, or font optimization was seen to have the most impact on patient comprehension.

**Conclusion:**

It is essential to present medication information in an optimal manner for patients. This can be done by standardizing the content, display, and format of medication labels to improve understanding and medication usage. Evidence-based design principles can, therefore, be used to facilitate the standardization of the structure of label content for both print and electronic devices. However, more research needs to be done on validating the implications of label content display to measure its impact on patient safety.

**Systemic review registration:**

PROSPERO CRD42022347510 (http://www.crd.york.ac.uk/prospero/).

**Supplementary Information:**

The online version contains supplementary material available at 10.1186/s13643-023-02413-z.

## Key points for decision-makers

• This study aims to review comparative evaluation studies on how medication label design can be optimized to improve patient understanding of medications and patient safety.

• It also discusses possible requirements for a new medication e-label standard to enhance the dissemination of tailored content via web and mobile apps.

• It revealed the importance of standardizing label content and display structure to incorporate into print and electronic formats.

**Supplementary Information:**

The online version contains supplementary material available at 10.1186/s13643-023-02413-z.

## Introduction

Medication labels provide vital health information about the investigational drug product, its preparation, dispensing, storage, and use. Although these labels are designed to assist patients’ understanding of their medications, over 50% of medication use errors, in terms of dosing, intervals, route of administration, etc., were seen to have occurred due to label miscomprehension by patients and their caregivers [1, 2]. According to the Institute of Medicine (IOM) report, inadequate labeling was cited as a significant cause of medication errors and adverse events due to improper understanding of instructions by the patient [3, 4]. Ultimately, instructions on these labels are increasingly important, especially if patients do not receive oral or written instructions from their providers on how to manage their medications appropriately. Patients with limited health literacy skills and managing multiple medication regimens are also at greater risk of experiencing medication errors due to misinterpretation of label instructions [4].

Additionally, revisions and updates to the approved labels occur quite often with about 400 to 500 product label changes occurring every year. Since drug labels are an essential tool to convey information about a medication’s indication, dosage, pharmacology, or adverse effects, it is important for patients to be made aware regarding any new information about a drug [5]. Disseminating these updates to patients can, ultimately, be time-consuming and, if not read, can lead to a potential risk to patient safety.

Variability in drug labeling can adversely affect a patient’s understanding of medication instructions [6, 7]. Previous studies have recommended some best practices, such as using plain language, improved formatting, and more explicit instructions for conveying prescription medication information to patients to improve patient safety [4, 6, 8–10]. Most recently, a study in 2018 reviewed the design of prescription drug labeling and educational materials. While it presented evidence supporting the best practices for prescription medication information, it did not evaluate how the design practices would impact patient outcomes and safety [10].

Currently, the US Food and Drug Administration (FDA) has developed a standard to regulate labeling content to meet better the prescribing practitioners’ needs (Physicians’ Labeling Rule, PLR) [11]. The PLR was designed to improve how health care practitioners access, read, and use medication labels by including specific sections, such as Highlights of Prescribing Information (Highlights), a Table of Contents (Contents), and the Full Prescribing Information (FPI) [12]. The central goal of the PLR is to provide structured labeling information that is easy to access, read, and use by both the FDA and the public to enhance the consistency in drug labeling [13]. Despite this, research has shown that patients still experience a lack of knowledge about the drugs prescribed to them and that increased adverse events can occur due to this lack of knowledge [8, 11].

**Fig. 1:**
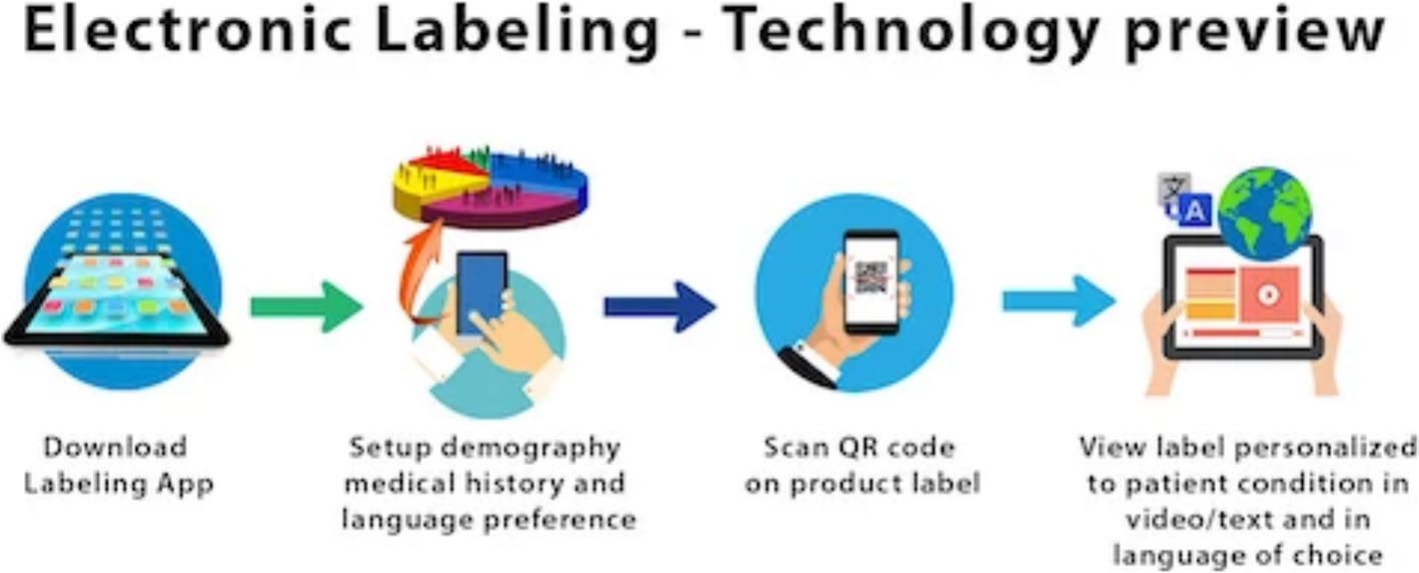
Electronic labeling—technology preview [14]

One possible way to improve the dissemination of patient medication information is through an electronic medication label, also known as an “e-label.” This e-label will be geared towards patients to enhance patient utilization and increase patient safety. The e-label can be accessed via electronic means, such as through a machine-readable QR code, barcode, or URL on the medication product itself (Fig. 1) [14].

The FDA does currently have a standard for drug information in electronic form using the HL7 standard Structured Product Labeling (SPL) [8]. This standard defines the content of human prescription drug labeling in an XML format [15]. SPL documents contain the content of labeling (all text, tables, and figures) for a product and additional computer-processable drug knowledge [15]. While the current SPL standard defines the content of human prescription drug labeling, it does not represent the optimal display format [11]. Effectively disseminating e-labels with content beyond text, such as graphics and video, will require standardization. The standard would describe both the structure of the content and display standards such as font size, language, typography, and other user interface standards for optimal display across electronic devices. This study, therefore, aims to systematically review comparative evaluation studies on how medication label design can be optimized to improve patient understanding of medications and patient safety. It also discusses possible requirements for a new medication e-label standard to enhance the dissemination of tailored content via web and mobile apps.

## Methods

This review was conducted following the Preferred Reporting Items for Systematic Reviews and Meta-Analyses (PRISMA) guidelines for reporting systematic reviews [16] (CRD42022347510; http://www.crd.york.ac.uk/prospero/).

Systematic computerized literature searches were performed in PubMed, Embase (Elsevier), Cochrane Central (EBSCO), CINAHL (EBSCO), and Web of Science (Thomson Reuters). The search was designed to identify studies that evaluated medication label design and how improvements in drug labeling can enhance patient knowledge. No date or language limits were applied, and no gray literature sources were examined. The search was performed on July 20, 2022. The search terms are listed in Appendix 1.

Inclusion criteria are English language articles that evaluate how the medication label design can be optimized to improve patient understanding of medications and safety. Studies to be included need comparative evaluations such as clinical study, comparative study, evaluation study, validation study, retrospective cohort study, prospective cohort study, randomized, controlled trial (RCT), cohort study, before-and-after study, or multicenter study. Articles that did not include a measure of health knowledge were excluded. Two blinded study authors independently reviewed the titles and abstracts of each article identified by the search. A consensus of all the authors settled any study selection disputes regarding whether studies obtained from the literature search met the inclusion/exclusion criteria to be included in this review.

Two blinded authors independently conducted the quality assessment and data extraction. The Covidence screening and data extraction tool for authors was used [17]. The quality assessment was conducted based on the recommendations of the Cochrane Risk of Bias Comparison: sequence generation, allocation concealment, blinding of participants, blinding of outcome assessors, incomplete outcome data, selective outcome, and other sources of bias. The following data were extracted: study identification, methods, population, intervention, and outcome variables. *p* values < 0.05 were considered statistically significant. A third author adjudicated disagreements between the two blinded authors.

## Results

**Fig. 2:**
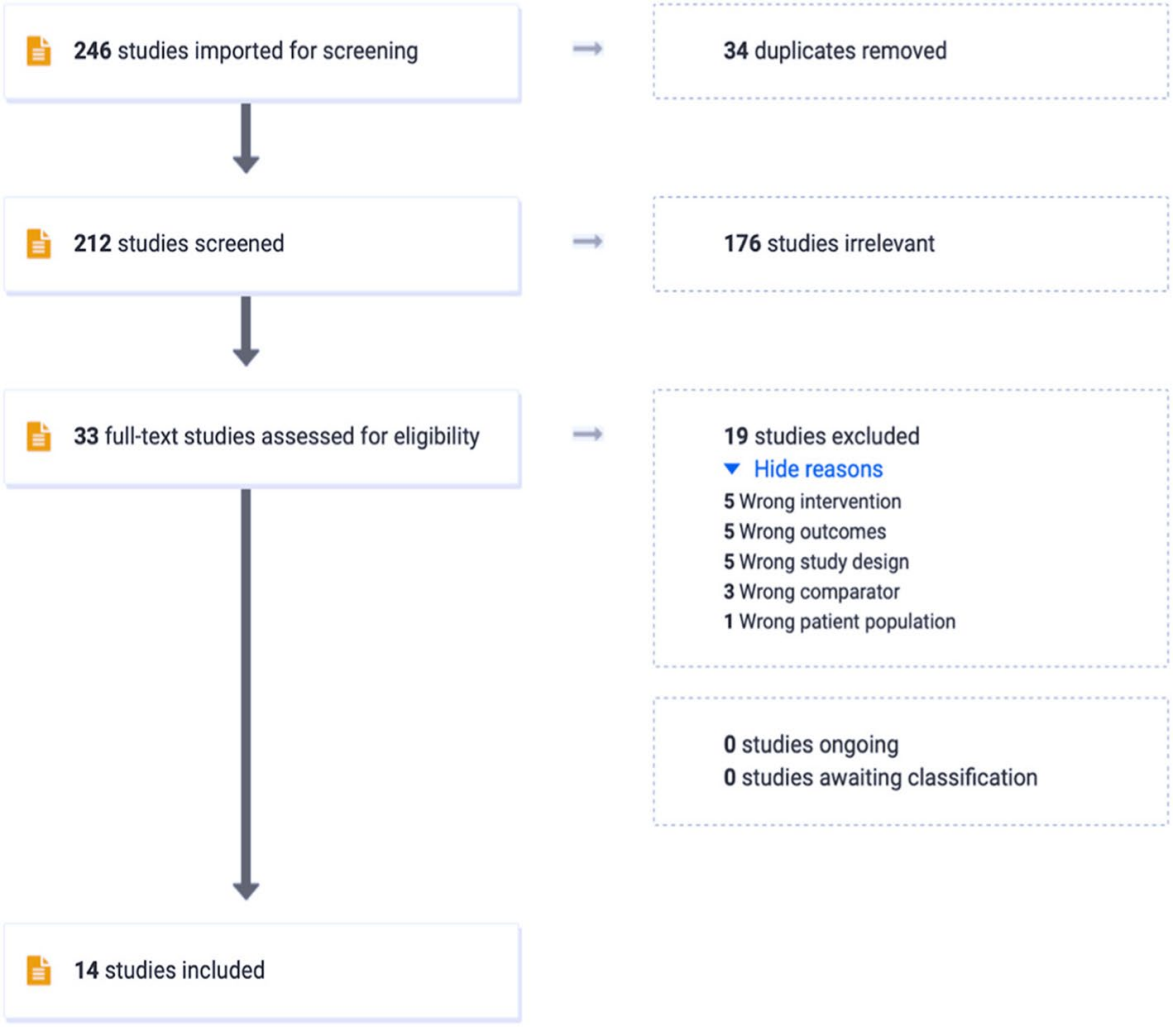
Preferred Reporting Items for Systematic Reviews and Meta-Analyses (PRISMA) flow diagram of the article search and review process

Of the 246 articles identified in the primary literature search, 212 were included in the title and abstract screening after removing duplicate articles. Of the 212 articles, 176 were excluded due to ineligibility. Following the exclusion, 33 articles were selected for the full-text review. Of those 33 articles, 19 studies were excluded due to not meeting the inclusion criteria. This resulted in 14 eligible studies identified for data abstraction, as presented in Fig. 2.

In this systematic review, the studies included were published from the years 1997 up to 2019. Nine of the studies were conducted in the USA, and one study was conducted in each of the following countries: Ireland, Australia, Hong Kong, Singapore, and South Africa. The included studies consisted of seven randomized control trials, three cross-sectional studies, one experimental study, one cohort study, one comparative study, and one structured interview design. These studies were all conducted via convenience, voluntary sampling, surveys, or clinic patients. The participants recruited from each of the studies consisted of the patient population, except for one study that also included participants involving physicians and pharmacists (Table 1). Of the included studies, 11 of them focused specifically on prescription medication labels while only 3 of the studies [18–20] focused only on over the counter (OTC) medication labels.

**Table 1:** Background information of included studies

Study ID	Country	Study design	Recruitment method	Participants
Chan 2013 [21]	Hong Kong	Non-randomized experimental study	Convenience sampling and voluntary participation	160
Bhansali 2016 [18]	USA	Cross-sectional, randomized between and within subjects experimental design	Convenience sampling	249
You 2011 [22]	USA	A comparative study, secondary analysis of a 3-arm clinical trial	Clinic patients	132
McCarthy 2013 [23]	USA	Randomized controlled trial	Convenience sampling	87
Sahm 2012 [24]	Ireland	Cross-sectional study	Clinic patients	94
Tong 2018 [19]	Australia	Qualitative research, cohort study	Voluntary	50
Tai 2016 [25]	USA	Randomized controlled trial	Voluntary	172
Friedman 1997 [20]	USA	Randomized controlled trial	Door-to-door interviews	2260
Law 2010 [26]	USA	Structured interview design	Convenience sampling	628 (444 patients, 115 pharmacists, 69 physicians)
Dowse 2005 [27]	South Africa	Randomized controlled trial	Clinic patients	87
Davis 2009 [9]	USA	Cross-sectional study	Clinic patients	359
Yin 2017 [28]	USA	Randomized controlled trial	Clinic patients	491
Wolf 2016 [29]	USA	Randomized controlled trial	Clinic patients	845
Malhotra 2019 [30]	Singapore	Randomized controlled trial	Survey	1414

Across the 14 included studies, the utilization of patient-centered language, in terms of the usage of explicit, deconstructed instructions, simplified text, numeric characters instead of words, the addition of pictograms/graphics and color/white space, or font optimization, were seen to have the most impact on patient comprehension (Table 2).

**Table 2:** Summary of studies: label design recommendations

Recommendations	Study
PCL: (plain language, simplified/explicit text)	- You 2011 [22], comparative study, *N* = 132 - McCarthy 2013 [23], RCT, *N* = 87 - Sahm 2012 [24], cross-sectional study, *N* = 94 - Davis 2009 [9], cross-sectional study, *N* = 359 - Friedman 1997 [20], RCT, *N* = 2260 - Law 2010 [26], structured interview design study, *N* = 628 - Tai 2016 [25], RCT, *N* = 172 - Wolf 2016 [29], RCT, *N* = 845
Addition of pictograms/graphics	- Chan 2013 [21], non-randomized experimental study, *N* = 160 - Dowse 2005 [27], RCT, *N* = 87 - You 2011 [22], comparative study, *N* = 132 - Malhotra 2019 [30], RCT, *N* = 1414 - Friedman 1997 [20], RCT, *N* = 2260 - Yin 2017 [28], RCT, *N* = 491
Color/white space optimization	- Law 2010 [26], structured interview design study, *N* = 628 - Malhotra 2019 [30], RCT, *N* = 1414 - Tong 2018 [19], cohort study, *N* = 50 - Yin 2017 [28], RCT, *N* = 491 - Wolf 2016 [29], RCT, *N* = 845
Font size optimization	- You 2011 [22], comparative study, *N* = 132 - Malhotra 2019 [30], RCT, *N* = 1414 - Law 2010 [26], structured interview design study, *N* = 628 - Wolf 2016 [29], RCT, *N* = 845
Bilingual text	- Malhotra 2019 [30], RCT, *N* = 1414
Congruent information	- Bhansali 2016 [18], cross-sectional study, *N* = 249

Of the eight studies that utilized patient-centered language, it was found that including explicit, deconstructed instructions and simplified text and numeric characters on prescription drug label instructions can improve patient comprehension [23, 24]. Specific features that were implemented to improve readability included using simple language for directions intended for 5th-grade reading skills (age range 10–11 years), inserting a table for times of administration, adding indications to the instructions, or adding a box for warnings and precautions. These features were incorporated to improve the convenience of finding information on the label [26].

Of the six studies that utilized pictograms/graphics for medication labels, it was found that for the pictograms to be effective, they should have direct connections with familiar aspects encountered in daily life. It was recommended that pharmaceutical pictograms be designed considering the following five features: familiarity, concreteness, complexity, meaningfulness, and semantic distance [21]. Further, in a population with limited reading skills, the inclusion of pictograms on medicine labels was found to positively influence the understanding of instructions and adherence [27]. Including pictograms on the labels was particularly valuable in communicating instructions on how to take medicine and in emphasizing the necessity of completing the course [27]. Furthermore, one study noted that patients found it most useful when the actual administration times were drawn in for each patient on a clock face diagram to avoid prolonged intervals between doses. Patients actively welcomed this pictogram as they found it to be instrumental in clarifying one of the most difficult features of taking multiple daily doses [27]. Including pictographic dosing diagrams was found to be especially helpful in preventing significant dosing errors [28].

In terms of layout, six studies recommended improving content layout via the utilization of color, white space, and font size optimization. Color backgrounds and white space were manipulated to improve the cosmetic appearance of the label. Recommendations also included using a bigger font size (larger font used for patient name, medication name, and dosage and directions in comparison to other components of the label) [26].

In terms of outcome measures, thirteen studies were found to directly measure the impact of patient understanding of their medication labels, and three studies were found to include a measure of patient safety in terms of medication adherence and dosing errors. All three studies correlated patient safety positively with the redesigned labels (Table 3). Of the included studies that evaluated how label reformatting impacted patient comprehension, 13 of the studies had structured interview assessments. In contrast, one study utilized the Short Test of Functional Health Literacy in Adults (STOFHLA) and Modified LaRue Tool (MLT) scores (Table 4). Most of the included studies utilized previous research efforts, patient feedback, and pilot testing methods to redesign medication labels in a more patient-centric format. Of the two studies that redesigned labels by utilizing pictograms to depict how to take medication, they obtained pharmaceutical pictograms from the US Pharmacopeia or the International Pharmaceutical Federation (FIP) (Table 4).

**Table 3:** Patient comprehension and safety

Study	Patient outcomes	Metric	Observations	Recommendations
Chan 2013 [21]	Comprehension	Guess ability; comprehension	- Mean and standard deviation of scores for all pharmaceutical pictograms were 64.8 and 17.1. The sign with the highest guess ability score was P5 (do not take if pregnant) (87.5), while the sign M3 (take 3 times a day with meals) had the lowest score (34.4) (*p* < 0.0001) - Prospective-user factors of the drug purchase experience, attention to drug labels, etc. had no significant effects on guessing performance (*p* > 0.05). The factors of “occupation,” “age,” and “education level” were found to significantly affect the guessing performance of participants (*p* < 0.0001) - The mean ratings for the sign feature evaluations for all the pharmaceutical pictograms were 53.5. (familiarity, SD = 9.7), 66.6 (concreteness, SD = 8.8), 66.3 (simplicity, SD = 8.2), 67.9 (meaningfulness, SD = 8.8), and 72.0 (semantic closeness, SD = 10.1), respectively	- User characteristics and sign features are critical for pharmaceutical pictograms if they are to effectively communicate messages to a user - Decisions about sign design that involve assumptions about familiarity should be considered together with prospective user factors - Pharmaceutical graphic designers need to emphasize the design of pharmaceutical pictograms, which have an obvious and direct connection with things encountered in daily life - To facilitate understanding of the meaning of a sign, the sign should provide a direct visualization aid so that there are links between what is illustrated in the sign and the function it is intended to represent
	Safety			
Bhansali 2016 [18]	Comprehension	14 item, 5-point Likert scale	Standard label: 4.1 ± 0.5 Label design 1: 3.9 ± 0.5 Label design 2: 4.1 ± 0.6	Place warning information at the end of the Drug Facts panel to improve patient information processing and understanding to enhance patient safety
	Safety			
You 2011 [22]	Comprehension	Comprehension	Standard label: 76% Design 1: 79% Design 2: 94% - Comprehension of the enhanced text + icon label was significantly higher than the standard and enhanced text-only labels - After adjusting for age, race/ethnicity, education, literacy, number of medications currently taken, and study site, comprehension of the enhanced text + icon label remained significantly higher than standard and enhanced text-only labels	Teratogen warning labels with easy-to-read messages and icons can significantly improve comprehension
	Safety			
McCarthy 2013 [23]	Comprehension			
	Safety	Dosing errors	- The Take-Wait-Stop label worked best on untangling one common aspect of instructions: the maximum daily dose message but did not significantly improve problems with errors related to the spacing of doses - Errors: Exceed max dose: (31.8% vs 14%) > 2 pills per dose: (2.3% vs 0%) Interval < 4 h: (20.5% vs 23.3%)	Future directions should improve how a Take-Wait-Stop approach can better communicate all aspects of proper dosing
Sahm 2012 [24]	Comprehension	Generalized estimating equation (GEE) model for correct interpretation of Rx label instructions	- PCL > > > standard for the more complex regimen, drug 3 (97% correct interpretation vs 79%; *p* = 0.02) In multivariate analyses - PCL > standard (not significant) - PCL > PCL + pictogram - Low literacy: PCL > standard (*p* = 0.06)	The PCL approach may improve patients’ understanding and use of their medication regimen
	Safety			
Tong 2018 [19]	Comprehension	User testing	Overall, all 4 alternative label formats supported consumers’ ability to find and understand key points The existing standard label was poorer concerning participants’ ability to find and understand key points. Factors such as perceived usability, color, design, content, and content ordering impacted consumer preferences Consumers often preferred the “Consumer Desires” or “Drug Facts” label over the TGA proposed format as the standardized OTC label	User testing of OTC labels and consumer feedback received as part of the testing process can assist in the refinement of OTC labeling to ensure that implemented policies are evidence-based
	Safety			
Tai 2016 [25]	Comprehension	MLT and STOFHLA score; analysis of covariance	- Participants using redesigned Rx labels showed significantly higher MLT scores than with current Rx labels both before (23.0 ± 2.3 vs 21.0 ± 2.4; *p* < 0.001) and after educational intervention (23.8 ± 1.7 vs 22.1 ± 3.1; *p* < 0.001) - With the use of analysis of covariance, intervention participants using redesigned label showed significant improvement in both MLT (23.1 ± 2.0 to 24.3 ± 1.0; *p* < 0.001) and STOFHLA (29.8 ± 7.5 to 31.5 ± 5.7; *p* = 0.011) scores, whereas intervention participants using current Rx label did not show significant improvement in either MLT (*p* = 0.530) or STOFHLA (*p* = 0.215) scores	- Use of a redesigned patient-centered Rx label and a label-focused educational intervention should be encouraged to improve Rx label comprehension and FHL - Simplifying communication with and confirming comprehension for all patients is one of the ways to improve primary care for people with limited health literacy
	Safety			
Freidman 1997 [20]	Comprehension	Open-ended answers were coded; statistical analysis was performed on close-ended questions	- Comprehension of the version of the symbol among high school non-graduates was significantly lower than the other versions - No meaningful differences were noted between the graphic-text version and the text-only version - There was a high rate of accuracy in participants (97%) selecting “see your doctor” on the label, (70%) selecting “read the package insert.” - Respondents clearly understood the label communication objectives for drug interactions. 80% indicated that one should speak to a doctor before stopping/starting prescription drugs - There was a high percentage of correct responses in the total population to close-ended questions: types of cholesterol, appropriate use of cholestyramine, preparation, and dosage	- Clear and comprehensible labeling of an OTC product is essential for establishing appropriate use among consumers and thereby maintaining safety and optimizing efficacy and compliance - Following a systematic approach to developing, revalidation, and testing drug labeling, key messages can be written so that consumers will understand the proper use of a new category of OTC medication for a complex chronic condition - Labeling must be able to convey the intended use of the product, provide adequate directions for its use, warn against potentially harmful effects, and provide instructions for an appropriate length of treatment and when to seek professional medical advice
	Safety			
Law 2010 [26]	Comprehension	Predominantly descriptive and also included correlational analysis	- The majority of patients, 366 (82.8%), preferred new labels over existing ones - About 40% of respondents reported the table of times for administration and indication (27.9%) as advantages of the new labels - Bigger font size and easiness of reading were other most preferred features, with 27.2 and 19.8% of responses	- Specific areas that could be changed include adding indications to the label, creating a timetable of medication administration, and creating a box for warnings - Change in the design of labels in addition to content may be welcomed by patients. These include font size increase and changes in placement and white space
	Safety			
Dowse 2005 [27]	Comprehension	Chi-squared tests were used to test for differences in the understanding of medicine instructions	- The average score for understanding was significantly better in the experimental group (E) (95.2%) than in the control group (C) (69.5%) - In the experimental group, the majority (72%) displayed a high level of understanding (> 90%) - Significant correlation was found between literacy and understanding in the control group but not in the experimental group < 50% understanding = C 22% vs E 0% 51–70% understanding = C 31.7% vs E 2.2% 71–90% understanding = C 31.7% vs E 26.1% 91–100% understanding = C 14.6% = E 71.7% *p* < 0.01	Policymakers and regulatory bodies in countries with a high incidence of inadequate literacy skills should pay particular attention to improving labeling practices and considering the inclusion of pictograms on selected medicine labels
	Safety	Adherence (pill count/volume + self-report schedule/instructions)	- A high adherence of greater than 90% was found for 54% of the experimental group, compared with only 2% of the control group - The presence of pictograms was found to contribute positively to both understanding of instructions and adherence	Desired levels of adherence should be specified for each disease and treatment
Davis 2009 [9]	Comprehension	Generalized estimating equation (GEE)	- 71% of adequate literacy, 84% of marginal literacy, and 93% of low literacy incorrectly interpreted one or more label instructions - Rates of correct interpretation were lowest for instructions that depicted frequency in hourly intervals or the number of times per day (53% and 61%, respectively) - Prescription instructions that gave periods or specific times were significantly less likely to be misinterpreted compared to those using the number of times per day - 23% of responses were coded as incorrect. 78% misunderstood instructions, with 37% misunderstanding a minimum of three labels - The interaction term for literacy and type of language used to depict drug frequency of use approached but did not reach statistical significance (ARR 0.91, 95% CI 0.85–1.01; *p* = 0.079)	- Use of precise wording on prescription drug label instructions can improve patient comprehension. Patient’s motivation, concentration, and comprehension might have been greater if they were reporting on their own medicine given by their physician for conditions they or their children had - It is likely that patient counseling will also be needed to address health literacy deficits
	Safety			
Yin 2017 [28]	Comprehension			
	Safety	Dosing accuracy	- 83.5% of parents made ≥ 1 dosing error (overdosing was present in 12.1% of errors), and 29.3% of parents made ≥ 1 large error (> 2 × dose) - Milliliter/teaspoon was associated with more errors than milliliter-only - Parents who received text-only (versus text and pictogram) instructions or milliliter/teaspoon (versus milliliter-only) labels and tools made more large errors	Including pictographic diagrams and milliliter-only units on labels and tools may be especially helpful in preventing large dosing errors
Wolf 2016 [29]	Comprehension	Chi-square tests; generalized linear models (GLM); generalized estimating equation (GEE)	- Patients receiving the PCL demonstrated slightly better proper use of their drug regimens at first exposure (76.9% vs 70.1%) and at 9 months (85.9% vs 77.4%) - The effect of the PCL was significant for English-speaking patients but not for Spanish speakers - Overall, the PCL label did not improve medication adherence; however, significant benefits from the PCL were found among patients with limited literacy and those with medications to be taken ≥ 2 times a day	- A simple modification to pharmacy-generated labeling, with minimal investment required, can offer modest improvements to regimen use and adherence, mostly among patients with limited literacy and more complex regimens - Even small increases in the proper use and adherence among subgroups of consumers might yield a cost–benefit
	Safety			
Malhotra 2019 [30]	Comprehension	Analysis of variance, chi-square tests, univariate and multivariate logistic regression models, univariate and multivariate negative binomial regression models	- The bilingual text label with pictograms (BLTP) had the highest proportions with complete and any understanding (40.1% and 76.5%) and the lowest median number of incorrect answers. Among those who could read English, we observed the highest proportion with complete understanding among those assigned to the BLTP label (59.5%), but the proportion with any understanding and the median number of incorrect answers among those with any understanding was largely similar across the four prototype PMLs - Those who are unable to read English, the median number of incorrect answers had lower median values in the BLT and BLTP labels than in the ET and ETP labels	Adding bilingual text with or without pictograms on prescription medication labels considerably improved elderly Singaporeans’ understanding of the labels, strongly suggesting its application in practice
	Safety

**Table 4:** Label table design process and evaluation

Study	Label	Label design process	Evaluation method
Chan 2013 [21]	Standard label	25 of the 81 pharmaceutical pictograms from the US Pharmacopeia were used	A questionnaire for capturing participants’ particulars was designed for use, and a sign evaluation sheet was prepared for participants to give ratings for the pictograms
	Label design 1		
	Label design 2		
Bhansali 2016 [18]	Standard label	Based on FDA guidelines	Based on the information processing constructs of the OTC Label Evaluation Process Model (LEPM)
	Label design 1	Based on the concepts of information congruency where the author defined uses, directions, and other information on the Drug Facts panel as a single chunk and placed congruent information after the warnings section	
	Label design 2	Based on the concepts of information congruency, directions, and other information on the Drug Facts panel as a single chunk and placed congruent information before the warnings section	
You 2011 [22]	Standard label	Current standard drug warning label for prescription containers	- Structured cognitive interview protocol developed by the research authors, along with actual prescription pill bottle containers - Rapid Estimate of Adult Literacy in Medicine (REALM)
	Label design 1	Patient-centered text on the labels was designed based on patient feedback and pilot testing	
	Label design 2	- Patient-centered text and icons developed based on patient feedback and pilot testing - Guidelines established by the International Organization for Standardization for the development and testing of universal icons	
McCarthy 2013 [23]	Standard label	Current standard label	Via structured interview and REALM
	Label design 1	Patient-centered, Take-Wait-Stop label developed based on previous research	
	Label design 2		
Sahm 2012 [24]	Standard label	Standard Rx label	Via structured interview and REALM
	Label design 1	A patient-centered label that specified explicit timing with standard intervals (morning, noon, evening, bedtime) or with mealtime anchors based on a Universal Medication Schedule (UMS)	
	Label design 2	Patient-centered label with instruction and a graphic aid to visually depict dose and timing based on a Universal Medication Schedule (UMS)	
Tong 2018 [19]	Standard label	- Existing standard label where the Medicine Information label was based on the design outlined in the Australian TGA consultation paper released in August 2014 and the Drug Facts label was based on the Drug Facts standardized OTC label format implemented in the USA	Interview consisting of the administration of a user testing questionnaire
	Label design 1	- Medicine Facts label: Navy blue print on white background; information split across two panels (of the box) - Based on a consumer-proposed label title and needs analysis findings - Aspects of previously implemented and tested written medicine information formats such as the US Drug Facts label and Australian Consumer Medicine Information formats were also integrated	
	Label design 2	- Consumer Desires label: Navy blue print on light blue background with warnings section presented in red using a pictograph system highlighting indications and contraindications using ticks and crosses - Based on suggestions proposed by consumers in the earlier needs analysis phase of this research	
Tai 2016 [25]	Standard label	Current Rx label standard	Short Test of Functional Health Literacy in Adults (STOFHLA) and Modified LaRue Tool (MLT) scores
	Label design 1	- Patient-centered label with additional “when to take this drug” feature, highlights of the drug name, quantity, refills, dose strength in yellow, highlights of direction and time to take meds in purple and packed with more warning information - Based on previous research and the US Pharmacopeia (USP) recommendations on labeling changes	
	Label design 2	- Patient-centered label with an educational intervention - Based on previous research and the US Pharmacopeia (USP) recommendations on labeling changes	
Freidman 1997 [20]	Standard label	Current label	A 2-part questionnaire to test comprehension
	Label design 1	- “Symbols” version—incorporated a bar graph showing dosage efficacy and universal symbols to highlight usage “do’s and don’ts” - Based on the treatment guidelines outlined in the Second Report of the NCEP Expert Panel on Detection, Evaluation, and Treatment of High Blood Cholesterol in Adults, the existing prescription labeling, input from physician experts - Label was analyzed by a software program, Flesch Reading Ease Formula, and revised	
	Label design 2	- Text-only version—the container label copy was presented in text format, without bar charts or symbols - Graphic-text version—presented container label copy in text form with a bar graph indicating the dosage efficacy, but without the universal symbols - Based on the treatment guidelines outlined in the Second Report of the NCEP Expert Panel on Detection, Evaluation, and Treatment of High Blood Cholesterol in Adults, the existing prescription labeling, input from physician experts - Label was analyzed by a software program, Flesch Reading Ease Formula, and revised	
Law 2010 [26]	Standard label	Current label	Patient interviews
	Label design 1	- Simple language for directions intended for 5th-grade reading skills (age range 10–11 years), inserting a table for times of administration, and adding indication to the instructions; color backgrounds and white space were manipulated to improve the cosmetic appearance of the label; bigger font size (for patient name, medication name, and dosage and directions); and addition of a box for warnings and precautions; size: 2.25″ × 3.75″ (5.715 × 9.525 cm) - The content of the label was based on 2008 California State law requirements for prescription drug labels (Sect. 4076) - The framework developed and used was categorized as content, cosmetic appearance, and convenience	
	Label design 2		
Dowse 2005 [27]	Standard label	Antibiotics that appear in the local Essential Drugs List were chosen based on level of usage	Recall and understanding of instructions were assessed using a series of structured questions; adherence was determined by self-reporting and “pill count”
	Label design 1	Pictograms used on the labels had been previously developed locally and tested in the South African population	
	Label design 2		
Davis 2009 [9]	Standard label	Current label	Via a structured interview; patient literacy was assessed using the Rapid Estimate of Adult Literacy in Medicine (REALM)
	Label design 1	- Variations in the frequency of use for the drug’s daily administration: number of times per day, hourly intervals, periods, or specific times; variations of the dosage instructions were used per drug, ranging from vague to most explicit - Three physicians and one pharmacist identified a typical dose for each medication, along with variations in the frequency of use for the drug’s daily administration	
	Label design 2		
Yin 2017 [28]	Standard label	Current label	- Parental health literacy was measured by using the Newest Vital Sign (NVS) - Dosing assessments (each subject participated in 9 total trials; they were asked to measure three dose amounts (2, 7.5, and 10 mL) using three tools (5- and 10-mL–capacity oral syringes and dosing cups with 15 mL as the largest marking)) - Survey to assess parents’ sociodemographic and health literacy
	Label design 1	- Text and pictogram instructions, “mL”-only label and tool; text and pictogram instructions, “mL/tsp” label and tool - Based on variations by type of instruction on the medication label and measurement units on the label and dosing tool	
	Label design 2	- Text-only instructions, “mL”-only label and tool and text-only instructions, “mL/tsp” label and tool - Based on variations by type of instruction on the medication label and measurement units on the label and dosing tool	
Wolf 2016 [29]	Standard label	Common template used by a national pharmacy chain at the time of trial initiation	- Demonstrated proper use of a prescribed drug in a patient’s regimen (questions) - Patient Medication Adherence Questionnaire (PMAQ) - Pill count - Rapid Estimate of Adult Literacy in Medicine (REALM) for English patients - Short Assessment of Health Literacy for Spanish Adults (SAHLSA) for Spanish Patients
	Label design 1	- Patient-centered label (PCL): changes included dose instructions that were written in UMS form, a graphic aid placed in the center of the label, below the instructions, to visually display the dose, a large, bolded font (12 point), increased whitespace - Followed only evidence-based practices for information format and content and supported by patient focus groups	
	Label design 2		
Malhotra 2019 [30]	Standard label	Standard English text label	Sixteen label-related questions regarding instructions indicated on the prescription medication labels
	Label design 1	- English text label with pictograms - Bilingual text label - Via the usage of the International Pharmaceutical Federation (FIP) pictograms	
	Label design 2	- Bilingual text label with pictograms - Via the usage of the International Pharmaceutical Federation (FIP) pictograms	

Only one study evaluated the impact of language choice on label comprehension. This study assessed the use of bilingual text on patient comprehension and found that adding bilingual text on prescription medication labels considerably improved understanding of the labels, especially among non-English speakers [30].

In terms of how to best communicate prescription label information to patients, only one study was found to discuss this topic. This study assessed how adding pharmacist counseling could improve patients’ comprehension of the medication label. This study found that incorporating a label highlighting critical educational components and a pharmacist-led education counseling session on the medication showed improved Rx label comprehension and functional health knowledge [25]. The label design was enough to solve the patient’s comprehension problem.

In terms of the quality of the studies, the quality assessment scores of the included studies are provided in Appendix 2.

### Bias assessment

**Fig. 3:**
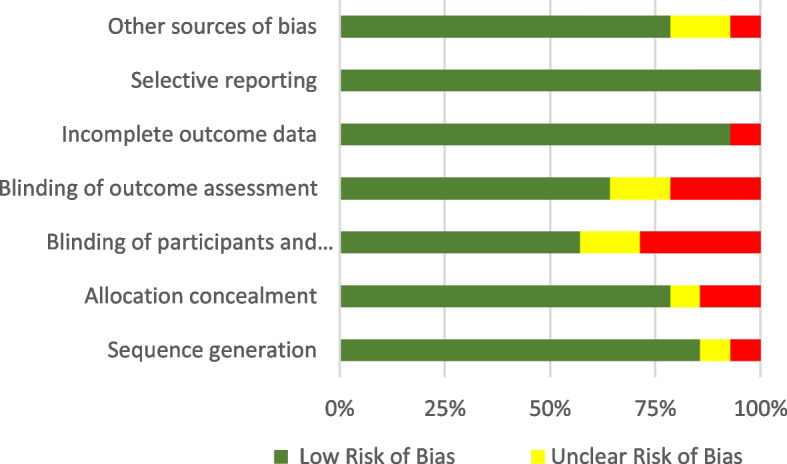
Bias evaluation

The Cochrane method was used to evaluate bias and is summarized in Fig. 3. Seven of the studies reviewed were deemed under the low-risk category for performance bias. The other seven of the studies had an unclear risk for performance bias as proper blinding of the personnel was not always guaranteed.

All the studies were deemed to be at low risk for attrition bias. A significant factor in determining the risk of bias was the inadequate reasoning for missing data. Nine reviewed studies were deemed low risk for detection bias, two at high risk, and three at unclear risk. Reporting bias was also considered to be at low risk across all studies. Three studies contained other potential sources of bias. These studies noted that some of the participants recruited work in healthcare [21], that most of the participants were young and had undergraduate or above education level [17], and that the majority of participants were female (93%) [27]. This was determined to meet the criteria for a high risk of bias due to the potential of increased health literacy and the low variability of the recruited participants. None of the studies were evaluated as having a high risk of bias due to a lack of information regarding funding sources or conflicts of interest.

## Discussion

This review evaluated how medication label content and display formats impact patient understanding and safety. It was found that incorporating specific design elements, such as adding visual aids, optimizing font or color, or patient-centered language, can improve label comprehension. Ultimately, specific format changes need to be addressed in order to ensure the proper identification of key information necessary for the safe and appropriate use of medications [31].

Due to contributing factors such as complex labeling language, confusing layout, or small font sizes, many patients may struggle to read and understand their medication labels. These may, in particular, impact populations such as the elderly, patients with low reading and health literacy, and patients with poor English proficiency [26]. According to previous studies, prescription labels that had been redesigned in a patient centric format were seen to be preferred over the current labels. Patients were, overall, found to favor labels that specifically highlighted the medication name, dosage, and directions, utilized increased font size, and color and white space optimization [19, 26, 28–31]. In regard to the usage of pictograms/graphics, previous studies found that graphics can improve comprehension if the illustrations and text are well-matched to each other and are appropriate and/or familiar to the background of the user [20–22, 27, 28, 30, 32]. Furthermore, among bilingual patients, it was reported that when provided with medication information in their native language, they had increased levels of understanding of their medications [30]. As a whole, these findings correlate with the results of this review in that the utilization of patient-centered language, pictograms/graphics, color/white space, or font optimization are seen to have the most impact on patient comprehension.

Therefore, it is of particular importance to pay attention to label layout and how to emphasize critical medication information to improve label readability. One way this can be done is through the utilization of e-labels. For, with e-labels, an adaptive used interface could be used. This interface could change the elements or layout on an electronic label to match the preferences of the individual user in terms of changing the font size or language. It could also provide label content in different formats, such as through low-language level text, pictograms, or video explanations.

For example, patients’ preferences for more graphical styles could display content using pictograms to users preferentially. Providing label content in these different formats can help improve label understanding by appealing to patients across all literacy levels.

Furthermore, label content could be tagged with medical terminology codes such that medication warnings could be more easily integrated into electronic medication reminder applications and drug-drug interaction warnings to patients. This tagging of alternative content descriptions could also be provided in graphical formats which could be used to improve the dissemination of tailored content to lower literacy or older adults via web or mobile apps.

Evidence-based recommendations on optimal content and display formats will, however, be necessary for new electronically encoded medication labels that go beyond the current SPL standard [33]. Furthermore, integrating standards beyond the healthcare field, such as IEEE (Institute of Electrical and Electronics Engineers), ISO (International Organization for Standardization), or ITU (International Telecommunication Union), could help with the interoperability of other applications, such as smart home monitoring, which could connect with healthcare provider apps and services [34, 35].

Certain challenges, however, should be noted with the utilization of electronically encoded medication labels. Limitations includes patient lack of access to a reliable electronic device or connectivity issues such as having access to Wi-Fi internet services to be able to access the label. Additionally, applying a new electronic standard will be a significant challenge to ensure that compliance with labeling accuracy, consistency, and traceability are met.

Key policy makers, such as the FDA and pharmaceutical industries, should, therefore, take note to consider how the above factors can impact patients and work to create standardized format and content practices for e-labels to provide patients with clear, concise, and accessible medication information. For developing a new electronic standard could improve connectivity and provider-to-patient communication, leading to better comprehension, safety, and patient experience.

In addition to formatting label display, the process of patient engagement should also be looked at to improve patient medication comprehension. A better process is needed to better engage with consumers to communicate medication information and improve label comprehension beyond the initial visit to a pharmacy. While the “teach-back” method, in which patients are asked to repeat instructions to demonstrate their understanding, is the currently recommended technique used to assess patient understanding of their medication label, it may not be enough for identifying potential errors in medication administration. Previous studies documented a gap between a patient’s ability to correctly state instructions and their ability to correctly demonstrate the correct number of pills to be taken daily. An enhanced approach is, therefore, needed to verify that patients can accurately describe and demonstrate how to take their medications [36–38]. However, more definitive studies are needed to inform practice standards on communicating medication information to consumers.

Furthermore, it is also important to consider how the content of information in a label can impact the benefit/risk perception of a drug regimen as this can affect a patient’s compliance with their drug therapy. Patients may perceive that the risk of the drug, such as in terms of side effects, is much more detrimental than the benefit of the drug. In that case, they may take the medication intermittently or stop taking it altogether, which can reduce the overall beneficial effect of the drug. Therefore, the way information is placed in a label, such as through more explicit or deconstructed text, is important as this can directly impact the patient’s perception of the drug and, in turn, their compliance/adherence to their regimen. A patient’s ability to understand the risk–benefit ratio may influence their medication adherence, and clinical trials may influence their choice to enroll in the study. There are particular problems in enrolling low-literacy patients in clinical trials [39, 40].

In order to provide patients with a balanced benefit/risk perception of their medication, it would be important to consider adding a section on benefits information to the label. This section will include more details on how the drug works, the relationship between the mechanism of action and the disease, and how the effects can be monitored [41, 42]. Adding this information on a label can provide patients with more information not only on the drug’s risks but also on its benefits and how it works to help them. This can lead to increased medication knowledge, ultimately influencing patient adherence/compliance [41].

One can, further, consider using human factors approaches to address problems for reducing medication errors, such as through label misinterpretation [43, 44]. Rather than reacting to medical errors, human factors analysis can consider processes, environments, interactions, and other resources to prevent them before they occur proactively. By incorporating human factors approaches in the designing process of medication labels, the labels can be designed in a way that is tailored specifically for patients, allowing the labels to be more patient centered. Ultimately, placing more emphasis on user-centered design can improve patient care and safety by providing another way that can be utilized to make labels more patient centric and improve their understating of label.

Certain limitations of this review should be noted. While an extensive search strategy was used, articles may have been missed using the previously described search methods. The scope of this review was also limited to only include articles utilizing a comparative design to assess an intervention’s effectiveness and articles written only in English. The keywords that were utilized in the literature search were not truncated, possibly resulting in the limitation of potentially relevant articles from other English-speaking countries.

Furthermore, sampling bias may have occurred in the 4 studies that utilized convenience sampling techniques rather than the gold standard of a randomized control trial as the study design. This may indicate that the results of these studies may not be generalizable to the greater population.

## Conclusion

This paper summarizes comparative studies that evaluated the design of a medication label to improve patient knowledge and safety. Most studies focused on usability and content, and few evaluated patient safety. This review further revealed the importance of standardizing label content and display structure to incorporate into print and electronic formats. It is essential to present medication information optimally for patients to improve understanding and medication usage. Evidence-based design principles can be used to standardize the structure of label content for both print and electronic devices. However, more research needs to be done on validating the implications of label content and display formats. The findings from this review reveal the importance of further assessment of the impact of label content and display, specifically on patient safety, and evaluation and validation of the label content display.

### Supplementary Information

**Additional file 1.**

## Data Availability

Data analyzed in this study are openly available at locations cited in the reference section.
