# Supplementary material for: Evaluation of the design and structure of electronic medication labels to improve patient health knowledge and safety: a systematic review
Source: Syst Rev. 2024 Jan 2;13:12. doi: 10.1186/s13643-023-02413-z (PMC10763215; doi:10.1186/s13643-023-02413-z)
Supplement: Supplementary file 1 — Additional file 1. [file 13643_2023_2413_MOESM1_ESM.docx]

Evaluation of the Design and Structure of Electronic Medication Labels to Improve Patient Health Knowledge and Safety: A Systematic Review

Sara Saif ^a,c^, Tien Bui ^b,c^, Gyana Srivastava ^c^, Yuri Quintana ^c^

^a^ Belmont University College of Pharmacy, 1900 Belmont Blvd, Nashville, TN, 37212, United States

E-mail: sara127s2hotmail.com

^b^ Massachusetts College of Pharmacy, 179 Longwood Ave, Boston, MA, 02115, United States

^c^ Division of Clinical Informatics, Beth Israel Deaconess Medical Center, 330 Brookline Avenue, Boston, MA, 02215, United States

**Appendix 1**

**Search Terms**

(Searches were conducted and uploaded on Covidence on Wednesday, July 20 2022)

**PubMed:**

**QUERY: (with study type) - 28 results**

("medication label" OR "drug label" OR "drug labeling/standard*" [mesh] OR "drug labeling/method*" [mesh]) AND ( "Health Literacy"[mesh] or "Health Literacy"[tiab] or "comprehension"[tiab] OR "Knowledge"[TI] OR "Understanding"[TIab] or "Patient Education as Topic*" [mesh] Or usability or Animations or illustrations or readability or font or "Presentation style" or "computer aided design" [mesh] or "drug design" [mesh] or "Audiovisual aids" [mesh] or "software design" or "format" [tiab] or "organization" [tiab])

AND

(Clinical Study [ptyp] OR Clinical Study[TIAB] OR Clinical Trial[ptyp] OR Clinical Trial[tiab] OR Comparative Study[TIAB] OR Evaluation Study[TIAB] OR Validation Studies[TIAB] OR control trial[TIAB] OR cohort[TIAB] OR RCT[TIAB] OR Randomized Control[TIAB] OR Randomized Controls[TIAB] OR Randomized Controlled[TIAB] OR retrospective study[TIAB] OR prospective study[TIAB] OR Before and after study[TIAB] or Multicenter Study[ptyp] or Multicenter Study[tiab] )

**Embase (Elsevier)**

**QUERY: 43 results**

(‘medication label’ OR ‘drug label’ OR ‘drug label*’/exp)

AND

 (‘Health Literacy’/exp OR Health Literacy:ab,ti or Comprehension:ab,ti OR Knowledge:ti OR Understanding:ab,ti or ‘Patient Education as Topic*’/exp Or usability or Animations or illustrations or readability or font or Presentation style or ‘computer aided design’/exp or ‘drug design’/exp or ‘Audiovisual aids’/exp or software design or format:ab,ti or organization:ab,ti )

AND

(Clinical Study:ab,ti OR Clinical Trial:ab,ti OR Comparative Study:ab,ti OR Evaluation Study:ab,ti OR Validation Studies:ab,ti OR control trial:ab,ti OR cohort:ab,ti OR RCT:ab,ti OR Randomized Control:ab,ti OR Randomized Controlled:ab,ti OR retrospective study:ab,ti OR prospective study:ab,ti OR Before and after study:ab,ti OR Multicenter Study:ab,ti or Randomized:ab,ti or trial:ab,ti)

**Cochrane Central (EBSCO):**

**(Using Title Abstract Search Feature)**

**QUERY : (with study type)- 42 Cochrane Reviews**

(medication label OR "drug label" OR "drug labeling/standard*" OR "drug labeling/method*" ) AND (“Health Literacy” or “Health Literacy” or “comprehension” OR “Knowledge” OR “Understanding” or “Patient Education as Topic*” Or usability or Animations or illustrations or readability or font or “Presentation style” or “computer aided design” or “drug design” or “Audiovisual aids” or “software design” or “format” or “organization”)

AND

(Clinical Study OR Clinical Trial OR Comparative Study OR Evaluation Study OR Validation Studies OR control trial OR cohort OR RCT OR Randomized Control OR Randomized Controls OR

Randomized Controlled OR retrospective study OR prospective study OR Before and after study or multicenter study)

**CINAHL (EBSCO):**

**QUERY: (with study type)- 82 results**

("medication label" OR "drug label" OR (MH ("drug labeling/standard+")) OR (MH ("drug labeling/method+"))) AND ( (MH ("Health Literacy+")) or TI ("Health Literacy") or AB (health literacy) or TI ("comprehension") or AB (comprehension) OR TI ("Knowledge") OR TI ("Understanding") or AB (understanding) or (MH ("Patient Education as Topic+")) Or usability or Animations or illustrations or readability or font or "Presentation style" or (MH ("computer aided design+")) or (MH ("drug design")) or (MH ("Audiovisual aids")) or "software design" or TI ("format") or AB (format) or TI ("organization") or AB (organization))

AND

(ZT (“clinical trial” OR “randomized controlled trial”) OR TI (Clinical Study OR Clinical Trial OR Comparative Study OR Evaluation Study OR Validation Studies OR control trial OR cohort OR RCT OR Randomized Control OR Randomized Controls OR Randomized Controlled OR retrospective study OR prospective study OR Before and after study or multicenter study) OR AB (Clinical Study OR Clinical Trial OR Comparative Study OR Evaluation Study OR Validation Studies OR control trial OR cohort OR RCT OR Randomized Control OR Randomized Controls OR Randomized Controlled OR retrospective study OR prospective study OR Before and after study or multicenter study))

**Web of Science (Thomson Reuters):**

**QUERY: (with study type)- 51 results**

(TI= (“medication label” OR “drug label” OR “drug labeling/standard” OR “drug labeling/method”) OR AB = (“medication label” OR “drug label” OR “drug labeling/standard” OR “drug labeling/method”))

AND

(TI = (Health Literacy OR comprehension OR knowledge OR understanding OR format OR organization OR Patient Education as Topic* OR usability OR Animations OR illustrations OR readability OR font OR Presentation style OR computer aided design* OR drug design OR Audiovisual aids OR software design) OR AB = (health literacy OR comprehension OR understanding OR format OR organization OR Patient Education as Topic* OR usability OR Animations OR illustrations OR readability OR font OR Presentation style OR computer aided design* OR drug design OR Audiovisual aids OR software design))

AND

(TI = (Clinical Study OR Clinical Trial OR Comparative Study OR Evaluation Study OR Validation Studies OR control trial OR cohort OR RCT OR Randomized Control OR Randomized Controls OR

Randomized Controlled OR retrospective study OR prospective study OR Before and after study or multicenter study) OR AB = (Clinical Study OR Clinical Trial OR Comparative Study OR Evaluation Study OR Validation Studies OR control trial OR cohort OR RCT OR Randomized Control OR Randomized Controls OR Randomized Controlled OR retrospective study OR prospective study OR Before and after study or multicenter study))

**Appendix 2**

**Study Quality Assessment Scores**

| **Study** | **Sequence generation** | **Sequence generation supporting text** | **Allocation concealment** | **Allocation concealment supporting text** | **Blinding of participants and personnel** | **Blinding of participants and personnel supporting text** | **Blinding of outcome assessment** | **Blinding of outcome assessment supporting text** | **Incomplete outcome data** | **Incomplete outcome data supporting text** | **Selective reporting** | **Selective reporting supporting text** | **Other sources of bias** | **Other sources of bias** |
| --- | --- | --- | --- | --- | --- | --- | --- | --- | --- | --- | --- | --- | --- | --- |
| Chan 2013 [18] |  | N/A |  | N/A |  | N/A |  | N/A | Low |  | Low |  | High | Most of the participants were young and had undergraduate or above education level;  Conducted using convenience sampling and voluntary participation |
| Bhansali 2016 [19] |  | N/A | Low | N/A |  | N/A | Low | N/A | Low |  | Low |  | Unsure | Some participants work in healthcare, so there might be bias regarding health literacy |
| You 2011 [20] |  | N/A |  | N/A | Unsure | N/A |  | N/A | Unsure | No information was provided regarding attrition. | Low | The study notes:  “We did not have information on patient’s health, and in particular whether they had experience with medication use.” | Low | Although there are biases, the study controlled for confounding variables: age, sex, race, low literacy, education, and the number of daily medications currently taken. The study corrected for the overestimation of variance. |
| McCarthy 2013 [21] |  | N/A |  | N/A |  | N/A |  | N/A | Low |  | Low |  | Low |  |
| Sahm 2012 [22] |  | N/A |  | N/A |  | N/A |  | N/A | Low |  | Low |  | Low |  |
| Tong 2018 [23] |  | N/A |  | N/A |  | N/A |  | N/A | Low |  | Low |  | Low |  |
| Tai 2016 [24] | Low | The simple computerized randomization scheme | Unsure |  | Unsure |  | Unsure |  | Low | Reported reasons for attrition/exclusions | Low |  | Low |  |
| Friedman 1997 [25] | Unsure |  | High |  | High |  | Low |  | Low |  | Low |  | Low |  |
| Law 2010 [26] |  | N/A |  | N/A |  | N/A |  | N/A | Low |  | Low |  | Low |  |
| Dowse 2005 [27] | High |  | Low |  | High | The intervention being tested in this study was a visual one, so neither the patients nor the researchers were blinded to the conditions | High |  | Low |  | Low |  | High | The majority of female participants in this study (93%); almost half of the cases in this study involved the pediatric preparation of amoxicillin suspension |
| Davis 2009 [28] |  | N/A |  | N/A |  | N/A |  | N/A | Low |  | Low |  | Low |  |
| Yin 2017 [29] | Low | The lead project coordinator developed the allocation sequence | Low | A random number generator, blocked by site, was used to assign subjects randomly; RAs remained blinded to assigned groups until after subject enrollment was finished | Low | RAs remained blinded to assigned groups until after subject enrollment was finished | High | RAs remained blinded to assigned groups until after subject enrollment was finished.  Once RAs began to administer dosing assessments, group assignments were evident from the labels and tools provided | Low |  | Low |  | Low |  |
| Wolf 2016 [30] | Low | Randomization via random number generator | Unsure |  | Unsure | Participants were blinded, and unsure of personnel. | Unsure |  | Low |  | Low |  | Low |  |
| Malhotra 2019 [31] | Low | Stratified block randomization | Low |  | Unsure |  | Unsure |  | Low |  | Low |  | Low |  |

Table 5. Quality Assessment Scores
